# Expatriate Nurses’ Experiences of Psychological Safety and Cultural Adaptation in Saudi Arabian Hospitals: A Qualitative Interview Study

**DOI:** 10.1155/jonm/6527310

**Published:** 2026-10-11

**Authors:** Asrar S. Almutairi, Rayhanah R. Almutairi, Hend N. Almutairi, Abdulaziz M. Alodhailah

**Affiliations:** ^1^ Department of Community and Psychiatric Mental Health Nursing College of Nursing Princess Nourah bint Abdulrahman University P.O. Box 84428 Riyadh 11671 Saudi Arabia pnu.edu.sa; ^2^ School of Archaeology and Anthropology College of Arts and Social Sciences The Australian National University Canberra 2601 Australian Capital Territory Australia anu.edu.au; ^3^ Department of Medical-Surgical Nursing College of Nursing King Saud University Riyadh 11451 Saudi Arabia ksu.edu.sa

**Keywords:** cultural adaptation, expatriate nurses, mentorship, nursing management, psychological safety, qualitative research, Saudi Arabia

## Abstract

**Aim:**

To explore how organisational support systems shape psychological safety and cultural adaptation among expatriate nurses working in Saudi Arabian hospitals.

**Background:**

Expatriate nurses make up a substantial share of the nursing workforce in Gulf health systems, and their integration is a recurring nurse management concern. Psychological safety, the shared belief that a team is safe for interpersonal risk‐taking, is a plausible but underexamined organisational mechanism linking management practice to expatriate nurse adaptation, voice and retention.

**Methods:**

Qualitative interview study with an interpretative phenomenological orientation, reported in accordance with the Consolidated Criteria for Reporting Qualitative Research (COREQ). Seventeen expatriate nurses (Philippines *n* = 7, India *n* = 5, Pakistan *n* = 3 and Egypt *n* = 2) were purposively recruited from three hospitals in Riyadh, Saudi Arabia (two government and one private), and each took part in one semistructured interview (45–75 min). Analysis followed a hybrid inductive–deductive procedure: Each transcript was first analysed idiographically and inductively, cross‐case patterns were then developed and only at that point were the patterns examined against Edmondson’s four psychological safety dimensions.

**Results:**

Three themes were constructed, each represented across the whole sample. Safety‐undermining workplace contexts comprised rigid hierarchies, implicit doubt about foreign credentials and unclear norms about how mistakes would be treated. Organisationally facilitated psychological safety was described in relation to formal mentorship, collegial assistance, hierarchical accessibility and explicit cultural competency work. Safety‐enabled adaptation captured accounts in which perceived safety accompanied question‐asking, experimentation, help‐seeking and authentic cross‐cultural engagement. Participants with access to structured mentorship (*n* = 8) described more consistent experiences of safety than those relying on informal or no support (*n* = 9); the design does not permit comparative or causal inference.

**Conclusion:**

Participants described psychological safety as an organisational condition that managers can act on, rather than an incidental by‐product of a pleasant workplace.

**Implications for Nursing Management:**

Nurse managers can treat psychological safety as something to be built and monitored: Train mentors, explicitly in safety‐building behaviours, make access to nursing leadership routine rather than nominal and locate cultural competency in everyday ward practice rather than in one‐off induction sessions.

## 1. Introduction

Psychological safety, defined by Edmondson as a shared belief that the team is safe for interpersonal risk‐taking, has become a central construct in research on organisational effectiveness, team learning, and healthcare quality [1, 2]. It describes the conditions under which employees will contribute ideas, ask questions, admit mistakes and seek assistance without fear of punishment, embarrassment or retaliation [3, 4]. Team‐level constructs of this kind, including psychological safety and team reflexivity, have been linked to team performance and innovative performance across sectors [5, 6].

In healthcare, psychological safety matters because the behaviours it permits are the behaviours that keep patients safe [7, 8]. Communication failure and diagnostic error remain leading contributors to preventable harm [9, 10], and low‐safety environments produce cultures of silence in which staff hesitate to report errors, raise concerns or offer alternative views [7, 11]. Psychological safety is therefore not only a wellbeing construct but an operational one, and it falls within the remit of nurse managers who set the tone of ward‐level interaction.

Expatriate nurses occupy a distinctive position in this literature. Gulf health systems, including Saudi Arabia, depend substantially on internationally recruited nurses, and nurse managers in these settings routinely lead teams drawn from many countries of origin. Internationally educated nurses face cultural difference, language barriers, credential‐recognition processes and the possibility of discrimination, all of which raise the interpersonal cost of speaking up relative to host‐country colleagues [12, 13]. They may be uncertain whether cultural mistakes will be treated as learning, whether their professional competence will be fairly appraised and whether they can safely acknowledge a learning need [12]. This matters for adaptation specifically because cultural integration requires exactly the behaviours that psychological safety underwrites: asking about unfamiliar social norms, admitting confusion and requesting clarification of professional expectations [11, 14].

Existing models of cultural adaptation have concentrated on the individual. Berry’s acculturation framework describes the strategies individuals adopt when negotiating two cultures [15], and Schlossberg’s transition theory describes the personal resources people draw on during major life transitions [16]. Neither specifies what an employing organisation should do. The nurse retention literature, in parallel, has concentrated on pay, staffing, workload and career development as determinants of intention to stay [17, 18]. Between these two literatures sit a question that nurse managers actually face: which everyday organisational practices make it safe enough for an internationally recruited nurse to ask, admit and contribute, and which do not.

Healthcare organisations managing expatriate nurses frequently rely on ad hoc arrangements rather than practices designed with any explicit mechanism in mind [7, 13, 19]. What is not known is how expatriate nurses themselves experience the organisational practices intended to support them, which of those practices they associate with feeling able to take interpersonal risks and what they identify as undermining that sense of safety. Addressing this gap matters because psychological safety, unlike national labour‐market conditions or salary structures, is largely within the control of nurse managers and nursing directors.

This study therefore aimed to explore how organisational support systems shape psychological safety and cultural adaptation among expatriate nurses in Saudi Arabian hospitals. Three questions guided the work: (i) which organisational practices and structures do expatriate nurses describe as undermining their sense of psychological safety; (ii) which organisational practices do they associate with a stronger sense of safety and (iii) how do they describe safety, once present, shaping the way they adapt to a new professional and cultural setting.

## 2. Methods

### 2.1. Design and Reporting

This was a qualitative interview study conducted within an interpretative phenomenological orientation. Reporting follows the Consolidated Criteria for Reporting Qualitative Research (COREQ), a 32‐item checklist for interview and focus group studies [20]; the completed checklist is provided as Supporting File 2 (COREQ checklist).

We describe the study as *phenomenologically oriented* rather than as a canonical interpretative phenomenological analysis (IPA), and Section 2.8 sets out precisely what was and was not taken from IPA. Orthodox IPA is idiographic, works with small and deliberately homogeneous samples and builds themes inductively without an a priori framework [21]. This study departs from that model in two respects that we consider methodologically consequential and therefore state openly: the sample was heterogeneous by design (four countries of origin, three hospitals, four clinical specialities), and an established theoretical framework was brought to bear on the data at a defined analytic stage. What was retained from IPA is its double‐hermeneutic commitment and its insistence that each participant’s account be understood in its own terms before any cross‐case claim is made.

### 2.2. Theoretical Framework

Edmondson’s psychological safety framework identifies four dimensions through which individuals experience safety in teams and organisations [1, 2]:

1. Voice and contribution: Speaking up with ideas, concerns or questions without fear of negative consequences.
2. Error tolerance: Acknowledging mistakes and learning needs without fear of punishment or blame.
3. Help‐seeking: Requesting assistance and admitting uncertainty.
4. Interpersonal risk‐taking: engaging authentically and with some vulnerability across difference.

The framework was used at a specified analytic stage (Section 2.8, Stage 3) as an organising lens for patterns already developed from the data, not as an a priori coding template applied to transcripts at first pass.

### 2.3. Epistemological Position

The study operates from a critical realist position: experiences of psychological safety are individually interpreted, yet are shaped by organisational structures and practices that exist independently of any one participant’s account. Both the subjective experience and the material arrangements of the workplace are treated as relevant to understanding adaptation.

### 2.4. Setting

The study was conducted in three hospitals in Riyadh, Saudi Arabia: two government hospitals and one private hospital, all of which routinely employ expatriate nurses. To preserve institutional confidentiality, the hospitals are referred to as Hospital A (government, approximately 800 nurses, formal mentorship programme), Hospital B (private, approximately 300 nurses, no formal mentorship programme) and Hospital C (government, approximately 400 nurses, informal mentorship arrangements only). Participants were drawn from all three sites: eight from Hospital A, seven from Hospital B and two from Hospital C. Participant characteristics are presented in Table 1.

**TABLE 1:** Study setting and participant characteristics (*N* = 17).

Characteristic	Details/distribution
Healthcare facility	Hospital A (government, approx. 800 nurses, formal mentorship programme): 8 (47.1%); Hospital B (private, approx. 300 nurses, no formal mentorship): 7 (41.2%); Hospital C (government, approx. 400 nurses, informal mentorship): 2 (11.8%)
Nationality	Philippines 7 (41.2%); India 5 (29.4%); Pakistan 3 (17.6%); Egypt 2 (11.8%)
Gender	Female 10 (58.8%); Male 7 (41.2%)
Age, years	Mean 35.4 (SD 8.7; range 24–51)
Nursing experience, years	Mean 8.1 (SD 4.2; range 2–18)
Tenure in Saudi Arabia, years	Mean 4.3 (SD 3.1; range 0.5–12)
Facility type	Government 10 (58.8%); Private 7 (41.2%)
Clinical speciality	Intensive care 5; emergency 4; paediatrics 4; medical–surgical 4
Access to mentorship	Formal programme 8 (47.1%); informal arrangements only 2 (11.8%); none 7 (41.2%)
Prior international experience	Yes 6 (35.3%); no 11 (64.7%)

*Note:* Characteristics are reported as sample‐level distributions only and are not linked to individual participant codes to reduce the risk of deductive disclosure. Mentorship access follows the arrangements available at each site: formal programme (Hospital A), informal arrangements only (Hospital C), none (Hospital B). SD = standard deviation.

### 2.5. Sampling and Recruitment

Purposive maximum‐variation sampling was used to obtain diversity in country of origin, gender, clinical speciality, tenure in Saudi Arabia, facility type and access to mentorship. Eligible participants were nurses of non‐Saudi nationality, recruited to work in Saudi Arabia from abroad, employed for at least 6 months at the participating hospital and able to be interviewed in English. Nurses who held a line‐management relationship with any member of the research team were not eligible.

Recruitment was deliberately structured so that no manager could identify who did or did not take part. Written recruitment notices describing the study and giving a study email address and mobile number were displayed in staff areas and circulated by a designated administrative staff member in each hospital who had no supervisory authority over nursing staff and no role in the research. Nurses who wished to take part contacted the research team directly and confidentially; the research team did not receive staff lists, and at no point did the team approach individual nurses through their line managers. Ward managers, unit heads and nursing directors were informed that the study was taking place at the site but were not told which staff had expressed interest, consented or been interviewed, and no participation records were shared with any employing institution. Interview appointments were arranged directly between the participant and the interviewer, at a time and place of the participant’s choosing, and were not logged in ward rosters or booking systems.

Three nurses who received the participant information sheet declined, citing incompatible work schedules. Seventeen nurses consented and completed an interview; none withdrew. Eight participants were recruited from Hospital A, seven from Hospital B and two from Hospital C.

### 2.6. Research Team and Reflexivity

Interviews were conducted by Abdulaziz M. Alodhailah and Asrar S. Almutairi, both PhD‐qualified nurse academics with prior training and experience in qualitative interviewing. Abdulaziz M. Alodhailah is a male Saudi national and a faculty member in medical–surgical nursing; Asrar S. Almutairi is a female Saudi national and a faculty member in community and psychiatric mental health nursing. Neither interviewer held a clinical, academic, supervisory or personal relationship with any participant before the study, and neither was employed by any of the participating hospitals. Participants were told at consent that the interviewers were university‐based researchers with no reporting line to hospital management and no role in performance appraisal, licensing or contract renewal.

The research team comprised the four authors. All four contributed to study design, coding, analysis and interpretation; Abdulaziz M. Alodhailah and Asrar S. Almutairi collected the data, and Rayhanah R. Almutairi co‐ordinated data management. No individuals outside the author group participated in coding or analysis, with the exception of the external peer debriefer described in Section 2.10, who questioned analytic decisions but did not code data or contribute to theme construction.

Two reflexive positions were identified at the outset and revisited throughout. First, as Saudi nurse academics, the interviewers were institutional insiders in the health system being described and cultural outsiders to the participants’ experience of it, a position that risked either normalising hierarchical practices participants found difficult or, conversely, over‐reading them as problematic. Second, team members with personal experience of international migration were identified during analysis, and their contributions to interpretation were explicitly discussed in team meetings so that personal narratives did not stand in for participants’ accounts. Both positions were recorded in individual reflexivity journals maintained throughout data collection and analysis, and both were raised deliberately in peer debriefing (Section 2.10). Where the interviewers noticed themselves supplying an explanation that a participant had not offered, this was recorded in the field notes and treated as an interpretive claim to be tested at the analysis stage rather than as data.

### 2.7. Data Collection

Each participant took part in one semi‐structured interview lasting 45–75 min (mean 58 min). No repeat interviews were conducted. Interviews were held in a private office or a quiet hospital room according to participant preference, and no one other than the participant and the interviewer was present.

A semi‐structured interview guide was developed for this study (Supporting File 1, interview guide). It used open‐ended questions allowing participants to describe experiences in their own terms, with probes covering five domains: initial experiences and workplace impressions; cultural and social integration; communication and language; organisational support systems; and adaptation strategies. The guide was refined through pilot interviews and iterative team discussion. Interviews were conducted in English, which was a working language for all participants but the first language of none; interviewers checked meaning by paraphrasing back and invited participants to correct any restatement.

All interviews were audio‐recorded with explicit consent and transcribed verbatim by a professional transcription service, bound by a confidentiality agreement, within 1 week of the interview. Field notes recording contextual observations, emotional tone and analytic reflections were completed immediately after each interview. Following transcription, each transcript was reviewed for completeness and returned to the respective participant for verification. Participants were invited to identify inaccuracies, clarify statements or provide additional information. Six participants (35%) returned minor corrections or clarifications, which the research team reviewed and incorporated where appropriate. Documentation of the verification process is retained by the research team and can be provided to the Editorial Office if required, subject to ethical and confidentiality requirements. Interviews were conducted between 1 June 2025 and 20 February 2026, following receipt of ethical approval on 21 May 2025.

Participant quotations are presented in English. Minor linguistic adjustments were made where necessary to improve grammatical clarity and readability. No substantive changes were made to participants’ accounts, no new information was introduced and identifying details were removed as described in Section 2.11.

### 2.8. Data Analysis

Analysis proceeded in four stages, using a hybrid inductive–deductive procedure of the kind described by Fereday and Muir‐Cochrane [22]. The sequencing matters for how the phenomenological orientation, the theoretical framework and the hybrid coding relate to one another, and is therefore reported in detail.

• Stage 1: idiographic, inductive case analysis. Each transcript was analysed on its own before any cross‐case work began, consistent with the idiographic commitment of IPA [21]. Each transcript was read and re‐read by at least two team members. A line‐by‐line descriptive coding identified meaningful units in the participant’s own terms (e.g., ‘healthcare practices differ’, ‘doctor–nurse hierarchy’, ‘feel comfortable asking questions’). Marginal annotations then recorded implicit meanings, contradictions and conceptual connections within that account. No a priori codes from Edmondson’s framework were available to coders at this stage, and coders were instructed to code what the participant said rather than what the study was about. Each transcript was summarised as a case‐level account before the next transcript was opened.
• Stage 2: inductive cross‐case pattern development. Once all 17 case‐level accounts existed, 87 preliminary codes were compared across cases and organised through iterative team discussion into 24 categories on the basis of conceptual similarity. Categories were then examined for hierarchical relationships and variation, producing 11 subordinate themes. Both steps remained data‐driven; theme labels at this stage used participants’ language.
• Stage 3: deductive framework mapping. Only at this point were the 11 subordinate themes examined against Edmondson’s four dimensions [1, 2]. Each subordinate theme was assessed for whether it spoke to one or more dimensions, to none of them, or to something the framework did not accommodate. This ordering was chosen specifically to limit the framework’s influence on theme construction: because the categories and subthemes already existed, the framework could organise them or fail to organise them, but could not generate them.
• Stage 4: integration and interrogation of framework fit. The 11 subordinate themes were synthesised into three overarching themes. The team then deliberately searched for material that the framework did not capture, on the reasoning that a good fit between data and framework is only meaningful if a poor fit was possible. Two areas were identified where Edmondson’s dimensions were insufficient: participants’ accounts of credential recognition and licensing, which concern institutional and regulatory positioning rather than team‐level interpersonal risk, and accounts of social inclusion outside clinical work, which participants linked to adaptation but not obviously to interpersonal risk‐taking at work. These are reported in Section 3 and returned to in Section 4.2, and they are the basis for our claim that the framework organises but does not exhaust the data.

Edmondson’s framework informed the conceptual orientation of the study but was not used as a predetermined coding template, and four features of the design were intended to keep the themes grounded in participants’ accounts. First, the interview guide was organised around adaptation and support (Section 2.7); none of its five domains corresponds to one of Edmondson’s dimensions, and participants were not asked to confirm, reject or comment on those dimensions. Second, all 87 codes, 24 categories and 11 subordinate themes were generated at Stages 1 and 2, before the framework was introduced, and were labelled in participants’ language. Third, the Stage 3 mapping allowed a subordinate theme to correspond to one, several or none of the dimensions, and two areas were only partly accommodated (Sections 3.2.2 and 3.3.2); accounts that extended beyond the framework were retained and considered in their own right. Fourth, the external peer debriefer was asked specifically to challenge the fit between framework and data (Section 2.10). The framework vocabulary in the final labels in Table 2 was applied at Stage 4, where the mapping held; the groupings beneath those labels predate it. Themes 1 and 2 are organised by workplace conditions and organisational practices rather than by dimension, and the closer correspondence in Theme 3 is acknowledged as a limitation in Section 4.4.

**TABLE 2:** Themes, subthemes, participant counts and psychological safety dimensions (*N* = 17).

Theme	Subtheme	Participants (*n*)	Psychological safety dimension(s)	Core mechanism
Theme 1. Safety‐undermining workplace contexts (*n* = 17)	1.1. Hierarchical constraints on professional voice	17	Voice and contribution (severely constrained)	Rigid hierarchy; limited access to senior staff
	1.2. Implicit messages about professional competence	15	Interpersonal risk‐taking; voice and contribution	Implicit critique of expatriate qualifications
	1.3. Uncertainty about mistake tolerance	14	Error tolerance (ambiguous)	Unclear norms about how error is treated

Theme 2. Organisationally facilitated psychological safety (*n* = 17)	2.1. Formal mentorship structures	8	All four dimensions	Structured relationship; protected time; trained mentor
	2.2. Collegial assistance and peer support	15	Help‐seeking; error tolerance	Informal senior‐colleague support
	2.3. Hierarchical accessibility initiatives	10	Voice and contribution	Leadership visibility and accessibility
	2.4. Explicit cultural competency integration	7	Interpersonal risk‐taking; voice and contribution	Organisational signalling that difference is valued

Theme 3. Safety‐enabled adaptation (*n* = 17)	3.1. Voice enabling faster learning	14	Voice and contribution	Direct questioning replaces inference from observation
	3.2. Error tolerance enabling experimentation	12	Error tolerance	Trial‐and‐error learning
	3.3. Help‐seeking enabling resource access	15	Help‐seeking	Access to guidance and information
	3.4. Interpersonal risk‐taking enabling authentic relationships	11	Interpersonal risk‐taking	Engagement beyond surface‐level interaction

*Note:* Theme‐level counts denote the number of participants contributing data to at least one subtheme within that theme; subtheme counts therefore do not sum to the theme‐level count because participants contributed to more than one subtheme.

Disagreements between analysts were resolved by a documented procedure rather than by seniority. All transcripts were coded independently by at least two team members. Where coders disagreed on the assignment or boundary of a code, the disagreement was recorded in the analytic memo and taken to the full team meeting, where both readings were argued against the transcript extract in question. Where the team could not agree, the extract was retained under both codes and carried forward to the next analytic stage rather than being forced to a decision, and the residual disagreement was raised in peer debriefing. Three disagreements were resolved in this way, all concerning whether accounts of hierarchical deference reflected constrained voice or a preferred professional norm; in each case, the resolution was to retain the participant’s own framing and to note the ambiguity in the theme description.

NVivo 12 (QSR International) was used for code management, memo writing and thematic organisation. The substantive analytic work was conducted through manual annotation and team discussion.

### 2.9. Sample Adequacy

Sample adequacy was judged using the concept of information power rather than data saturation [23], since saturation is tied to methodologies whose logic differs from the one used here and is inconsistently applied [23]. Information power was assessed against Malterud and colleagues’ five criteria. The aim was narrow (organisational conditions bearing on one construct) rather than broad; sample specificity was high since all participants were internationally recruited nurses working in the same city and health system; established theory was applied, which reduces the number of participants required; the quality of dialogue was strong, with interviews of 45–75 min conducted by experienced interviewers and verified by participants; and the analysis strategy was cross‐case rather than single‐case narrative. On these grounds, 17 participants were judged to hold sufficient information power for the study aim. Consistent with this judgement, no new codes or subordinate themes were generated from the final three interviews.

### 2.10. Trustworthiness

Several strategies were used to support credibility, dependability and confirmability.

#### 2.10.1. Engagement With the Setting

The team was in contact with the three sites for approximately nine months (June 2025 to February 2026) across recruitment, data collection and verification, which supported familiarity with organisational context. We do not claim ethnographic immersion: the data corpus consists of interviews and researcher field notes, not sustained observation of clinical work.

#### 2.10.2. Triangulation

Interview data were considered alongside researcher field notes at the analysis stage. Analyst triangulation was achieved through independent double coding of all transcripts, with the disagreement procedure described in Section 2.8. Theoretical triangulation involved reading the data against psychological safety theory and against acculturation and transition frameworks [15, 16].

#### 2.10.3. Member Checking

Six purposively selected participants, representing a range of nationalities, genders, tenure and facility types, reviewed an eight‐page preliminary thematic summary. All six confirmed that the themes were recognisable in their own experience. Two suggested refinements to theme wording were incorporated.

#### 2.10.4. Peer Debriefing

An external qualitative researcher (PhD‐level, specialising in phenomenological methods) met the team on four occasions during analysis, questioning analytic decisions, proposing alternative interpretations and challenging the fit between framework and data. Sessions were documented in the analytic memo.

#### 2.10.5. Negative Case Analysis

Two participants (P7, P14) described unexpectedly smooth adaptation. Both had prior international experience and specialist credentials in demand. These cases were used to define boundary conditions for the themes: adaptation was not uniformly difficult, and prior international experience and specialist status appeared to buffer the difficulties others described.

#### 2.10.6. Audit Trail

Preliminary codes, categorisation decisions, theme development rationale, team discussion notes and revisions to interpretation were documented throughout.

### 2.11. Ethical Considerations

Ethical approval was granted by the Institutional Review Board of King Saud University (Ref: KSU‐HE‐25‐1254, 21 May 2025). The study adhered to the Declaration of Helsinki, International Council of Nurses guidance and Saudi research ethics requirements. Administrative permission was obtained from each participating hospital.

#### 2.11.1. Informed Consent

Written informed consent was obtained from every participant before data collection, following an explanation of the study purpose, procedures, the researchers’ backgrounds and institutional independence, confidentiality arrangements, and the right to decline any question or withdraw at any time without giving a reason and without any consequence for employment. Participant information was provided in participants’ first languages where possible. Consent explicitly covered the publication of anonymised verbatim quotations.

#### 2.11.2. Protection From Workplace Consequences

Expatriate nurses may feel exposed when discussing managers, errors or discrimination, particularly where employment and residency status are linked. Several safeguards were built into the design. Recruitment bypassed the line‐management chain entirely (Section 2.5), so no manager knew who participated. Interviews were held away from clinical areas at times chosen by participants. Participants were told at consent that nothing they said would be reported to their employer, that no findings would be returned to any participating hospital in a form that could identify a unit or an individual, and that they could skip any question or stop the interview at any point. Contact details for independent counselling services were provided to all participants, and interviewers were trained to recognise and respond to distress and to pause or end an interview if needed.

#### 2.11.3. Confidentiality and Protection Against Deductive Disclosure

Nationality, workplace, speciality and quotation together could identify an individual, particularly within the smaller nationality groups. This risk was taken seriously in reporting. Participants were assigned codes (P1–P17). Hospitals are identified only as A, B and C, and no hospital is described in a way that would permit identification. Critically, the identifiers reported in Table 1 are reported only as sample‐level distributions and are never linked to individual participant codes: no quotation in Section 3 is attributed to a combination of participant code, nationality, hospital, speciality and tenure. Where a participant’s own words name a country of origin, that country is retained because removing it would render the account unintelligible, but no other identifier is attached to that participant anywhere in the manuscript, and the corresponding hospital and speciality are not reported. Identifying details of individuals, units and institutions were removed from transcripts before analysis.

#### 2.11.4. Data Protection

All data were stored on encrypted, password‐protected devices accessible only to the research team. Identifiable information was excluded from analysis files and from all outputs.

## 3. Results

Analysis produced three themes. Table 2 summarises the themes, subthemes, the number of participants contributing to each and the psychological safety dimensions to which each subtheme was mapped at Stage 3 of the analysis.

### 3.1. Overview

Every participant described workplace conditions that made interpersonal risk feel costly. A smaller group described organisational practices that reduced that cost, and those participants also described adapting differently: asking rather than inferring, trying rather than adhering and engaging rather than withdrawing. The three themes are presented in that order because participants themselves narrated them that way, as a starting condition, an intervening set of practices and a consequence.

### 3.2. Theme 1: Safety‐Undermining Workplace Contexts (*n* = 17)

All participants described structural and relational features of their workplaces that eroded psychological safety, leaving them uncertain whether adaptation mistakes would be tolerated, whether their competence would be fairly evaluated or whether they could safely acknowledge learning needs.

#### 3.2.1. Hierarchical Constraints on Professional Voice (*n* = 17)

Participants described rigid professional hierarchies that constrained their ability to contribute ideas, ask clarifying questions or raise concerns. Several contrasted these with flatter structures in their countries of origin and described the resulting dissonance as a signal that voice was unwelcome:

‘In my previous position in India, if you had a concern about patient care, you could speak to the doctor. Here, the hierarchy is very rigid. You don’t speak to the doctor unless directly addressed. This makes you feel you don’t have a voice, that your concerns won’t be heard. This affects how you communicate, makes you very careful, very reserved’ (P3).

‘The organisational culture here emphasises obedience and following hierarchy strictly. As a nurse from another country, this is amplified, you’re already uncertain about your position, and the hierarchy makes you feel even smaller. You don’t ask questions. You follow orders. This limits your ability to contribute to patient care decisions’ (P2).

Participants described this constraint as most acute early in their tenure, when uncertainty about workplace norms was highest. It should be noted that hierarchical deference was not uniformly experienced as a deprivation: in three cases, coders disagreed over whether an account described constrained voice or a professional norm the participant endorsed, and these accounts have been retained with the participant’s own framing (Section 2.8).

#### 3.2.2. Implicit Messages About Professional Competence (*n* = 15)

Fifteen participants described subtle or explicit messages, questioning their professional competence on the basis of foreign credentials, accent or cultural difference, and linked these to a reduced willingness to take interpersonal risks:

‘When I came here, I had to redo parts of my nursing license and training. This sent a clear message: ‘We don’t fully accept your credentials from Pakistan.’ Even though the training was relatively brief, it communicated that there was doubt about my foundational competence. This doubt stayed with me. It made me second‐guess myself, hesitant to contribute ideas’ (P5).

‘Colleagues sometimes made comments about my accent, implying I wasn’t communicating clearly. Even when I was speaking clearly, the implication was “You’re different, perhaps less competent because of that difference.” When you’re uncertain about whether people respect your competence, you don’t speak up. You don’t take risks’ (P11).

Participants described a position in which competence had to be demonstrated rather than assumed. Two elements of these accounts sit outside Edmondson’s team‐level dimensions: credential recognition and licensing are institutional and regulatory processes rather than interpersonal ones, yet participants described them as carrying an interpersonal message. This is one of the two areas of framework misfit identified at Stage 4 of the analysis, and it is returned to in Section 4.2.

#### 3.2.3. Uncertainty About Mistake Tolerance (*n* = 14)

Fourteen participants described not knowing whether a mistake would be read as ordinary learning or as evidence of unfitness, and located this uncertainty in the early employment period when learning‐related error is most likely:

‘When I made mistakes early on, I wasn’t sure whether people would understand this as normal, I’m new, I’m learning, or whether they would think “See? Foreign nurses aren’t as competent as Saudi nurses.” This uncertainty made me very anxious about making mistakes. I wouldn’t try new approaches because I was terrified of failing and that failure being attributed to me being foreign’ (P6).

Participants described the practical consequence as risk aversion: where the response to error was unpredictable, the safer course was to avoid situations in which error was possible, which is also to avoid the situations in which adaptation occurs.

### 3.3. Theme 2: Organisationally Facilitated Psychological Safety (*n* = 17)

All participants described at least one organisational practice that increased their sense of safety, although which practices were available differed by site. These accounts clustered around four practices.

#### 3.3.1. Formal Mentorship Structures (*n* = 8)

All eight participants contributing to this subtheme were recruited from Hospital A, the only site with a formal mentorship programme. They described a structured relationship with a designated mentor, protected time for it and scheduled opportunities for guidance, and they linked that relationship to safety across all four dimensions.

Participants described mentors as making the permissibility of voice explicit:

‘My mentor made it clear from the first meeting: “Your job is to ask questions. I want to hear your concerns. If something seems wrong, tell me. Your perspective matters.” This explicit permission to speak up changed everything. I felt that my voice was valued, not just tolerated’ (P8).

Mentors were described as normalising error:

‘When I made mistakes early on, my mentor explained: “Everyone makes mistakes when learning. I made the same mistakes when I first came here. This is how we learn. The important thing is that you’re learning.” This message, that mistakes are expected and acceptable, gave me confidence to keep trying, to take risks’ (P10).

Participants described explicit invitations to seek help:

‘My mentor said explicitly: “If you don’t understand something, ask. If you feel confused, come to me. There’s no stupid question.” This permission to ask for help was crucial. It made seeking assistance feel safe, not weak or shameful’ (P14).

And they described mentors modelling their own uncertainty:

‘My mentor talked about her own struggles when learning systems in different places. She wasn’t perfect; she admitted confusion and mistakes. This authenticity made me feel safer being authentic, admitting what I didn’t know, engaging genuinely’ (P1).

Across these accounts, participants attributed effectiveness to five features of the mentoring arrangement: explicit mentor training in psychological safety and cultural competency; protected time (participants described 2–4 hours weekly); a defined mentor role; regular scheduled meetings; and visible organisational recognition that mentoring was legitimate work. These are participants’ attributions and were not independently verified against programme documentation.

#### 3.3.2. Collegial Assistance and Peer Support (*n* = 15)

Fifteen participants described informal support from experienced colleagues, which built safety in the absence of any formal programme. Colleagues were described as providing practical guidance, encouragement, translation help and social inclusion:

‘My colleagues, senior nurses who had been here longer, took me under their wings. They explained procedures, answered questions patiently, including me in social conversations. This informal mentorship made me feel I wasn’t alone, that I could ask questions without shame, that mistakes would be understood as learning’ (P7).

The obverse was also described. Five participants reported dismissive or unwelcoming colleagues and linked this to prolonged adaptation difficulty and reduced safety. Notably, several accounts in this subtheme concerned social inclusion outside clinical work, which participants tied to adaptation but which does not map cleanly onto interpersonal risk‐taking at work; this is the second area of framework misfit identified at Stage 4.

#### 3.3.3. Hierarchical Accessibility Initiatives (*n* = 10)

Ten participants described organisational practices that made senior nurses, managers and physicians more accessible:

‘Our new nurse manager started an open‐door policy, explicitly saying “Come talk to me anytime, about anything.” She also began having informal coffee times where staff could chat with her informally. This accessibility conveyed: “Your concerns matter, I want to hear from you.” It didn’t eliminate the hierarchy, but it made it less rigid, more human’ (P3).

Participants described these initiatives as bearing chiefly on voice: they created routes for raising concerns that did not require going through formal channels that participants expected to suppress them. Participants distinguished between managers who announced accessibility and those who enacted it, and it was the second group whose practices they described as changing anything.

#### 3.3.4. Explicit Cultural Competency Integration (*n* = 7)

Seven participants described facilities in which cultural competency appeared in training, leadership communication, policy review and routine discussion rather than only at induction:

‘We had cultural competency training where leaders explicitly discussed the importance of diverse perspectives, including those from different countries. The message was: “Cultural difference is valued here, not just tolerated.” This explicit message changed the psychological climate substantially’ (P4).

Participants described the operative element as the explicitness of the organisational message that difference, and the learning behaviours associated with it, were expected rather than tolerated.

### 3.4. Theme 3: Safety‐Enabled Adaptation (*n* = 17)

Participants who described a stronger sense of safety also described adapting differently. The four subthemes below correspond to the four dimensions of the framework, which is unsurprising given the Stage 3 mapping and should not be read as independent confirmation of the framework.

#### 3.4.1. Voice Enabling Faster Learning (*n* = 14)

Fourteen participants linked feeling able to ask questions with faster acquisition of cultural and professional knowledge:

‘Because I felt safe asking questions, I could learn rapidly. I asked about norms I didn’t understand, cultural practices that seemed strange, professional expectations. Within weeks, I had clarity that might have taken months if I’d tried to figure it out by observation alone. The ability to ask questions directly accelerated my adaptation tremendously’ (P16).

A contrasting account:

‘I tried to figure out cultural norms by observation. This took much longer. I made mistakes interpreting things. I couldn’t ask directly because I wasn’t sure if my questions would be welcomed. My adaptation took much longer because I didn’t have voice’ (P11).

#### 3.4.2. Error Tolerance Enabling Experimentation (*n* = 12)

Twelve participants described how the belief that mistakes would be treated as learning allowed them to try unfamiliar approaches:

‘When I believed mistakes would be treated as learning, I could try things. I tried different ways of approaching doctors. I tried different communication styles. Some worked, some didn’t. I learned through trying rather than through paralysing fear. This trial‐and‐error learning accelerated my adaptation’ (P8).

Where error tolerance was ambiguous, participants described adhering to established patterns rather than exploring alternatives.

#### 3.4.3. Help‐Seeking Enabling Resource Access (*n* = 15)

Fifteen participants described help‐seeking as the route to practical support, information and guidance:

‘I could ask for help. I asked about procedures, asked for explanations, asked for introduction to people who could help me. This help‐seeking enabled me to rapidly access resources and information I needed. People who couldn’t seek help stayed confused much longer’ (P3).

Participants described help‐seeking as particularly consequential for unfamiliar bureaucratic and administrative procedures, where observation alone yielded little.

#### 3.4.4. Interpersonal Risk‐Taking Enabling Authentic Relationships (*n* = 11)

Eleven participants described safety as a precondition for engaging authentically across cultural difference:

‘When I felt psychologically safe, I could be myself. I could ask about cultural practices that seemed strange to me, joke about cultural misunderstandings, acknowledge my confusion. This authenticity built genuine relationships. People who felt less safe maintained more formal, protective boundaries. The relationships stayed at a surface‐level’ (P1).

### 3.5. Variation Across the Sample

Participants with access to a formal mentorship programme (*n* = 8, all from Hospital A) described experiences of safety more consistently and across more dimensions than those with informal arrangements only or none (*n* = 9). Two participants (P7, P14) described comparatively smooth adaptation despite limited formal support; both had prior international experience and specialist credentials, which suggests that individual positioning can substitute to some degree for organisational provision. These patterns are descriptive of this sample. The study was not designed to compare groups, participants were not randomly allocated to mentorship and hospital, speciality and prior experience are confounded with mentorship access; no inference about the effect of mentorship should be drawn from these counts.

## 4. Discussion

### 4.1. Principal Findings

Expatriate nurses in this study described a workplace baseline in which interpersonal risk was expensive: hierarchy limited voice, credential and accent‐based signals cast doubt on competence, and norms about error were unclear. Against that baseline, four organisational practices were associated with a stronger sense of safety: structured mentorship, collegial assistance, enacted leadership accessibility and cultural competency treated as routine practice. Participants who described feeling safer also described adapting through asking, experimenting, help‐seeking and authentic engagement rather than through observation and withdrawal.

The contribution here is not that psychological safety matters, which is established, but that expatriate nurses locate it in identifiable, manageable practices. Three of the four practices participants described are directly within a nurse manager’s discretion.

### 4.2. Relation to Existing Literature

#### 4.2.1. Psychological Safety Theory

The framework organised the data well but did not exhaust it. Two areas fell outside its team‐level scope. First, credential recognition and licensing processes are institutional and regulatory, yet participants read them as interpersonal messages about their competence and carried that reading into ward‐level interaction. Second, social inclusion outside clinical work was linked by participants to adaptation without obviously constituting interpersonal risk‐taking at work. Both suggest that in expatriate contexts, psychological safety is shaped by conditions upstream of the team, a point the original framework was not designed to address [1, 2].

#### 4.2.2. Cultural Adaptation Literature

Berry’s acculturation framework and Schlossberg’s transition theory both centre individual strategy and resource [15, 16]. Participants’ accounts here point to organisational conditions as prior: the strategies these models describe presuppose that asking, admitting and engaging are safe enough to attempt. This does not displace individual‐level models so much as specifying a condition on which they depend.

#### 4.2.3. Nurse Workforce and Retention Literature

Studies of nurse retention have concentrated on compensation, workload, staffing and career development [17, 18]. Psychological safety appears in participants’ accounts as a distinct concern that these determinants do not capture; participants who described adequate pay and manageable workload still described being unable to speak. This is consistent with, though not evidence for, the proposition that safety is a separable determinant of expatriate nurse retention.

#### 4.2.4. Mentorship Literature

Mentorship in nursing has been studied largely as knowledge transfer, socialisation and career guidance [24, 25]. What participants emphasised was different: mentors who said explicitly that questions were expected, who narrated their own early mistakes and who invited help‐seeking in words. The safety‐building function of mentorship appears in these accounts as at least as consequential as its content‐transfer function, which has implications for what mentors are trained to do [26, 27].

#### 4.2.5. Hierarchy

Participants’ accounts of hierarchy as a constraint on voice align with findings from primary care teams [28] and with the wider literature on participative and destructive leadership styles [29, 30]. The distinction participants drew between announced and enacted accessibility is worth noting: an open‐door policy that is stated but not practised was described as changing nothing.

### 4.3. Implications for Nursing Management

The implications below are drawn from participants’ accounts in a single‐time‐point qualitative study and should be read as directions for managerial attention and for evaluation, not as evidence that any intervention produces a given outcome.

#### 4.3.1. At Team Level

Ward managers can make the permissibility of asking explicit rather than implicit. Participants repeatedly described the operative moment as a mentor or manager stating in words that questions were part of the job. Managers can also make their own response to error predictable: participants’ difficulty was less with punitive responses than with not knowing what the response would be.

#### 4.3.2. At Mentorship‐Programme Level

Where mentorship programmes exist, participants attributed their effect to features that are specifiable and auditable: mentor training that names safety‐building behaviours, protected time, a defined role, regular meetings and organisational recognition of mentoring as work. Programmes that assign mentors without these features are, on participants’ accounts, unlikely to do what programmes with them did. Existing peer support can be formalised on the same basis, with experienced internationally recruited nurses recognised and resourced as mentors [31].

#### 4.3.3. At Organisational Level

Nursing directors can examine whether hierarchical arrangements exceed what clinical authority requires. Participants distinguished necessary clinical decision authority, which they did not contest, from communicative deference that prevented raising concerns. Access to leadership needs to be enacted and observable rather than declared. Cultural competency work belongs in routine practice, policy review and leadership communication rather than in induction alone [32, 33]. Organisations should also recognise that credential recognition and licensing processes, which are administrative from an institutional viewpoint, are read by internationally recruited nurses as judgements about their competence; how these processes are explained is a manageable variable.

#### 4.3.4. At Policy Level

Regulators and workforce planners responsible for international recruitment might consider that the conditions supporting safe practice by internationally recruited nurses are organisational as well as regulatory, and that credential‐recognition requirements carry a signalling function beyond their administrative purpose.

#### 4.3.5. For Evaluation

Nurse managers implementing any of the above should build in measurement. Psychological safety is measurable with validated instruments, and organisations introducing mentorship or accessibility initiatives are positioned to evaluate them rather than assume their effect.

### 4.4. Strengths and Limitations

#### 4.4.1. Strengths

The study addresses a population that is central to Gulf health systems and under‐represented in the psychological safety literature. All transcripts were independently double‐coded with a documented disagreement procedure; transcripts were verified by all participants and themes by a subgroup; and the analysis deliberately searched for material the framework could not accommodate rather than only for material it could.

#### 4.4.2. Limitations

The sample of 17 nurses from four countries of origin, recruited from three hospitals in one city, is small, and some subgroups are very small (Pakistan *n* = 3, Egypt *n* = 2). Participants were also unevenly distributed across sites (Hospital A *n* = 8, Hospital B *n* = 7, Hospital C *n* = 2), so no comparison between facilities is warranted, and accounts of informal mentorship arrangements rest on two participants. Findings must not be read as representative of expatriate nurses in Saudi Arabia, of any national group within that population or of Saudi hospitals generally. Nationality is reported as a sample characteristic, and no claim is made that any account is typical of nurses from a given country; differences between the accounts of participants from different countries were not analysed and could not have been with subgroups of this size.

The design captures a single time point, so accounts of adaptation over time rely on retrospective recall and are subject to recall bias and post hoc coherence. Participants self‐selected in response to recruitment notices, which may have favoured those with stronger views in either direction, and the sample cannot include the perspectives of nurses who left Saudi Arabia, whose accounts of failed adaptation are absent by construction.

Interviews were conducted in English, which was a working language but a first language for none of the participants. Some nuance was almost certainly lost, and participants may have expressed themselves less fully than in their first languages. This is a limitation of the data itself, not only of its interpretation.

Only expatriate nurses were interviewed. Saudi colleagues, mentors and nurse managers were not, so accounts of hierarchy, mentorship and cultural competency initiatives are single‐perspective, and mentorship programme features are participants’ attributions rather than verified programme characteristics. Field notes were treated as a supporting data source; the study involved no sustained observation of clinical practice, so we do not claim ethnographic evidence for what participants described.

Finally, the framework‐informed analysis carries a known risk: once Edmondson’s four dimensions were applied at Stage 3, subsequent presentation is organised by them, and the correspondence between the four subthemes of Theme 3 and the four dimensions should be read as a product of that organisation rather than as independent corroboration. The staged sequence and the search for misfit at Stage 4 were designed to limit this risk but cannot eliminate it.

### 4.5. Future Research

Quantitative work using validated psychological safety instruments could establish whether the associations participants described hold at scale, and whether psychological safety predicts retention independently of pay and workload. Intervention studies could test structured mentorship, leadership accessibility initiatives and embedded cultural competency programmes against defined safety and retention outcomes. Longitudinal designs would establish whether safety precedes adaptation as participants described, or develops alongside it. Multi‐perspective designs including Saudi colleagues, mentors and nurse managers would test whether the practices participants identified are recognised as such by those enacting them, and comparative work across other Gulf and international destination countries would establish the boundaries of transferability. Extension to other internationally recruited health professionals would establish whether these mechanisms are specific to nursing.

## 5. Conclusion

Expatriate nurses in three Riyadh hospitals described a workplace in which speaking up, admitting error and asking for help carried real interpersonal cost and identified specific organisational practices that lowered that cost: structured mentorship with trained mentors and protected time, supportive senior colleagues, leadership access that was enacted rather than announced, and cultural competency treated as everyday practice. Where participants described feeling safe, they described adapting by asking and trying rather than by observing and withholding. These are participants’ accounts from a single‐time‐point qualitative study and do not establish that these practices produce adaptation or retention. What they do indicate is that psychological safety, for this workforce, is described as located in practices that nurse managers already control and is therefore available as an object of management attention and of evaluation rather than as a by‐product to be hoped for.

## Author Contributions

Asrar S. Almutairi and Abdulaziz M. Alodhailah conceptualised the study and designed the research. Asrar S. Almutairi and Abdulaziz M. Alodhailah conducted the interviews. Rayhanah R. Almutairi co‐ordinated data management and contributed to coding and analysis; Asrar S. Almutairi and Hend N. Almutairi contributed to data interpretation. Abdulaziz M. Alodhailah provided methodological guidance and critically revised the manuscript.

## Funding

This study was funded by the Ongoing Research Funding Program (ORF‐2026‐1146), King Saud University, Riyadh, Saudi Arabia.

## Disclosure

All authors read and approved the final version.

## Ethics Statement

Ethical approval was obtained from the Institutional Review Board of King Saud University (KSU‐HE‐25‐1254, 21 May 2025). Administrative permissions were obtained from participating healthcare institutions in Riyadh, Saudi Arabia. All participants received written and verbal information explaining the study purpose, procedures, voluntary participation and confidentiality. Written informed consent was obtained prior to data collection. All procedures were conducted in accordance with the ethical standards of the institutional research committee and with the 1964 Declaration of Helsinki and its later amendments.

## Consent

Written consent for the publication of anonymised verbatim quotations was obtained from all participants as part of the informed consent process. No identifiable personal data are included in this manuscript.

## Conflicts of Interest

The authors declare no conflicts of interest.

## Supporting Information

Additional supporting information can be found online in the Supporting Information section.

## Supporting information


**Supporting Information 1** Supporting File 1: Interview guide. The semistructured interview guide used for all 17 interviews, comprising open‐ended questions and probes across five domains: initial experiences and workplace impressions, cultural and social integration, communication and language, organisational support systems and adaptation strategies.

**Supporting Information 2** Supporting File 2: COREQ checklist. The completed 32‐item Consolidated Criteria for Reporting Qualitative Research (COREQ) checklist for this study.

## Data Availability

The interview transcripts are not publicly available because they contain potentially identifiable participant information and are subject to the confidentiality conditions of the approved ethics protocol. Deidentified methodological materials, including the interview guide, coding framework, selected anonymised coding examples and appropriate audit‐trail extracts, may be made available to the Editorial Office or reviewers upon reasonable request, subject to ethical and confidentiality requirements.
