# Supplementary material for: Expatriate Nurses’ Experiences of Psychological Safety and Cultural Adaptation in Saudi Arabian Hospitals: A Qualitative Interview Study
Source: J Nurs Manag. 2026 Oct 11;2026:6527310. doi: 10.1155/jonm/6527310 (PMC13655501; doi:10.1155/jonm/6527310)
Supplement: Supplementary file 1 [file JONM-2026-6527310-s001.docx]

**Supplementary File 1**

**Supplementary File 1. Interview guide.** The semi-structured interview guide used for all 17 interviews, comprising open-ended questions and probes across five domains: initial experiences and workplace impressions; cultural and social integration; communication and language; organisational support systems; and adaptation strategies.

**Semi-Structured Interview Guide: Organisational Support and Cultural Adaptation in Saudi Arabia**

***Purpose***

This interview guide accompanies the manuscript “Expatriate Nurses’ Experiences of Psychological Safety and Cultural Adaptation in Saudi Arabian Hospitals: A Qualitative Interview Study.” It was developed to elicit expatriate nurses’ accounts of workplace psychological safety and cultural adaptation, structured around Edmondson’s psychological safety framework (voice and contribution, error tolerance, help-seeking, and interpersonal risk-taking) and five thematic domains established during instrument development. The guide was refined through pilot interviews and iterative team discussion prior to use in the main study.

***Administration notes***

Interviews were conducted individually, in English, in a private and confidential setting selected according to participant preference (university office or hospital quiet room). Each interview was audio-recorded with participant consent and lasted approximately 45–75 minutes. Interviewers used the domain structure below flexibly, following participants’ narratives and using probes only where topics were not spontaneously raised.

***Opening script (read to participant before recording begins)***

“Thank you for agreeing to speak with me today. I’m interested in your experiences working as a nurse from another country here in Saudi Arabia — what has helped you settle in, what has made it harder, and how you’ve been supported (or not) by the people and systems around you. There are no right or wrong answers; I am interested in your own experience. You can decline to answer any question, and you may stop the interview at any time without any effect on your employment. With your permission, I will record our conversation so I can focus on listening rather than note-taking.”

## Domain 1 — Initial Experiences and Workplace Impressions

Establishes baseline psychological safety perceptions upon arrival.

**1.** Tell me about when you first started working here. What was that first period like for you?

*Probe: What stood out most in those first few weeks?*

**2.** When you had a question or concern early on, what did you do?

*Probe: Did you feel comfortable raising it? Why or why not?*

**3.** How did you come to understand what was expected of you professionally in this workplace?

## Domain 2 — Cultural and Social Integration Experiences

Explores barriers and facilitators to psychological safety arising from cultural difference.

**4.** Can you describe a time when a cultural difference affected how you worked or how you were treated?

*Probe: How did you make sense of that at the time?*

**5.** How do you think your nationality or background has affected how colleagues or supervisors see your professional competence?

**6.** What has helped you feel accepted, or not accepted, as part of the team?

## Domain 3 — Communication and Language Challenges

Examines the help-seeking and voice dimensions of psychological safety.

**7.** Tell me about a time you needed to ask for help or clarification. What happened?

*Probe: What made it easy or difficult to ask?*

**8.** Have you ever held back from speaking up about a patient care concern? What led to that?

**9.** How comfortable do you feel raising concerns with senior colleagues or physicians? Has that changed over time?

## Domain 4 — Organisational Support Systems

Directly addresses the organisational mechanisms hypothesised to build psychological safety.

**10.** What formal or informal support has been available to you since joining — for example, mentorship, orientation, or peer support?

*Probe: If you had a mentor: what did they do, or say, that made a difference?*

*Probe: If you did not have a mentor: how did you find the guidance and information you needed?*

**11.** How accessible do you find your managers or supervisors when you need to raise something?

**12.** Has your workplace done anything specifically related to cultural differences or supporting international staff? What was that like?

**13.** When you have made a mistake, what happened? How was it handled?

*Probe: What did that experience teach you about whether mistakes are treated as learning opportunities here?*

## Domain 5 — Adaptation Strategies and Resilience

Examines how (or whether) psychological safety enables effective adaptation.

**14.** Looking back, what has helped you adapt most?

**15.** Is there anything you do differently now, compared to when you first arrived, in terms of speaking up, asking for help, or admitting mistakes?

**16.** What would you want healthcare organisations to know about supporting nurses coming from other countries?

## Closing

**17.** Is there anything else about your experience that we haven’t covered that you think is important for me to understand?

“Thank you again for your time and openness. Your transcript will be shared with you afterward so you can review it and let us know if anything needs correcting. Please let me know if you have any questions.”
