# Supplementary material for: Expatriate Nurses’ Experiences of Psychological Safety and Cultural Adaptation in Saudi Arabian Hospitals: A Qualitative Interview Study
Source: J Nurs Manag. 2026 Oct 11;2026:6527310. doi: 10.1155/jonm/6527310 (PMC13655501; doi:10.1155/jonm/6527310)
Supplement: Supplementary file 2 [file JONM-2026-6527310-s002.docx]

**Supplementary File 2**

**Supplementary File 2. COREQ checklist.** The completed 32-item Consolidated Criteria for Reporting Qualitative Research (COREQ) checklist for this study.

**COREQ (Consolidated Criteria for Reporting Qualitative Research): 32-Item Checklist**

*Completed per Tong A, Sainsbury P, Craig J. Consolidated criteria for reporting qualitative research (COREQ): a 32-item checklist for interviews and focus groups. Int J Qual Health Care 2007;19(6):349–57.*

| No. | Item | Guide question | Reported in manuscript |
| --- | --- | --- | --- |
|  | **Domain 1: Research team and reflexivity** |  |  |
|  | ***Personal characteristics*** |  |  |
| 1 | Interviewer/facilitator | Which author(s) conducted the interview or focus group? | Section 2.6. Interviews were conducted by A.M.A. and A.S.A. The author contribution statement records the same two authors. |
| 2 | Credentials | What were the researcher’s credentials? | Section 2.6. Both interviewers are PhD-qualified nurse academics. |
| 3 | Occupation | What was their occupation at the time of the study? | Section 2.6. Both are university faculty members: A.M.A. in medical-surgical nursing, A.S.A. in community and psychiatric mental health nursing. Neither was employed by any participating hospital. The research team comprised the four authors; no non-author participated in coding or analysis apart from the external peer debriefer (Section 2.10). |
| 4 | Gender | Was the researcher male or female? | Section 2.6. A.M.A. is male; A.S.A. is female. |
| 5 | Experience and training | What experience or training did the researcher have? | Section 2.6. Both interviewers had prior training and experience in qualitative interviewing. |
|  | ***Relationship with participants*** |  |  |
| 6 | Relationship established | Was a relationship established prior to study commencement? | Section 2.6. Neither interviewer held a clinical, academic, supervisory or personal relationship with any participant before the study. Nurses in a line-management relationship with any team member were ineligible (Section 2.5). |
| 7 | Participant knowledge of the interviewer | What did participants know about the researcher? | Sections 2.6 and 2.11. Participants were told at consent that the interviewers were university-based researchers with no reporting line to hospital management and no role in performance appraisal, licensing or contract renewal, in addition to written and verbal information on study purpose, procedures, confidentiality and participant rights. |
| 8 | Interviewer characteristics | What characteristics were reported about the interviewer, e.g. bias, assumptions, interests? | Section 2.6. Two reflexive positions are stated with what each risked: institutional insider to the health system and cultural outsider to the participants’ experience; and team members with personal experience of international migration, whose contributions to interpretation were explicitly discussed. Both were recorded in individual reflexivity journals and raised in peer debriefing. Interviewer-supplied explanations noticed during interviews were logged in field notes as interpretive claims to be tested, not treated as data. |
|  | **Domain 2: Study design** |  |  |
|  | ***Theoretical framework*** |  |  |
| 9 | Methodological orientation and theory | What methodological orientation was stated to underpin the study? | Sections 2.1–2.3. A qualitative interview study conducted within an interpretative phenomenological orientation, not a canonical interpretative phenomenological analysis (IPA); the two departures from orthodox IPA (heterogeneous sample by design; application of an established framework) are stated explicitly, as is what was retained (double hermeneutic; case-level analysis before cross-case comparison). Edmondson’s psychological safety framework is applied at a defined analytic stage (Section 2.8, Stage 3). Critical realist epistemological position. |
|  | ***Participant selection*** |  |  |
| 10 | Sampling | How were participants selected? | Section 2.5. Purposive maximum-variation sampling for diversity in country of origin, gender, clinical specialty, tenure, facility type and mentorship access. Eligibility criteria stated. |
| 11 | Method of approach | How were participants approached? | Section 2.5. Written recruitment notices in staff areas giving a study email address and mobile number, circulated by a designated administrative staff member at each site with no supervisory authority over nursing staff and no research role. Nurses contacted the research team directly and confidentially; no staff lists were provided to the team and no nurse was approached through a line manager. |
| 12 | Sample size | How many participants were in the study? | Section 2.5 and Table 1. N = 17, distributed as Hospital A 8, Hospital B 7, Hospital C 2. |
| 13 | Non-participation | How many people refused to participate or dropped out? Reasons? | Section 2.5. Three nurses who received the participant information sheet declined, citing incompatible work schedules. None of the 17 who consented withdrew. Numbers initially expressing interest and formally screened were not recorded and are therefore not reported. |
|  | ***Setting*** |  |  |
| 14 | Setting of data collection | Where were the data collected? | Section 2.7. A private office or a quiet hospital room according to participant preference, away from clinical areas and at times chosen by participants (Section 2.11). |
| 15 | Presence of non-participants | Was anyone else present besides the participants and researchers? | Section 2.7. No one other than the participant and the interviewer was present. |
| 16 | Description of sample | What are the important characteristics of the sample? | Table 1: site, nationality, gender, age, nursing experience, tenure in Saudi Arabia, facility type, clinical specialty, mentorship access, prior international experience. Data collection period 1 June 2025 to 20 February 2026 (Section 2.7). Characteristics are reported as sample-level distributions only and are never linked to individual participant codes (Section 2.11). |
|  | ***Data collection*** |  |  |
| 17 | Interview guide | Were questions, prompts, guides provided? Was it pilot tested? | Section 2.7. Semi-structured guide developed for this study across five domains, refined through pilot interviews and iterative team discussion. Full guide provided as Supplementary File 1. |
| 18 | Repeat interviews | Were repeat interviews carried out? | Section 2.7. No repeat interviews were conducted; each participant took part in one interview. |
| 19 | Audio/visual recording | Did the research use audio/visual recording? | Section 2.7. All interviews were audio-recorded with explicit consent. |
| 20 | Field notes | Were field notes made during and/or after the interview? | Section 2.7. Field notes recording contextual observations, emotional tone and analytic reflections were completed immediately after each interview. |
| 21 | Duration | What was the duration of the interviews? | Section 2.7. 45–75 minutes; mean 58 minutes. |
| 22 | Data saturation | Was data saturation discussed? | Section 2.9. Sample adequacy is assessed using information power (Malterud et al. 2016) rather than data saturation, because saturation belongs to a methodological tradition whose logic differs from the one used here. All five information-power criteria are addressed: narrow aim, high sample specificity, application of established theory, quality of dialogue, and cross-case analysis strategy. Consistent with that judgement, no new codes or subordinate themes were generated from the final three interviews. |
| 23 | Transcripts returned | Were transcripts returned to participants for comment and/or correction? | Section 2.7. Transcripts were returned to all 17 participants; six (35%) returned minor corrections or clarifications, which were incorporated. |
|  | **Domain 3: Analysis and findings** |  |  |
|  | ***Data analysis*** |  |  |
| 24 | Number of data coders | How many data coders coded the data? | Section 2.8. All transcripts were independently coded by at least two team members. Disagreements were recorded in the analytic memo and argued at full team meetings against the transcript extract, not resolved by seniority; where the team could not agree, the extract was retained under both codes and carried forward. Three such disagreements occurred, all concerning whether accounts of hierarchical deference reflected constrained voice or an endorsed professional norm. |
| 25 | Description of the coding tree | Did authors provide a description of the coding tree? | Section 2.8 describes the progression from 87 preliminary codes to 24 categories, 12 subordinate themes and three overarching themes. Table 2 sets out the resulting structure: three themes, eleven subthemes, participant counts and the psychological safety dimension to which each subtheme was mapped. No separate visual coding tree is provided. |
| 26 | Derivation of themes | Were themes identified in advance or derived from the data? | Section 2.8. Derived from the data. Stage 1 was idiographic and inductive, with each transcript analysed and summarised on its own before the next was opened and no a priori framework codes available to coders. Stage 2 developed cross-case patterns inductively. Edmondson’s framework entered only at Stage 3, applied to subordinate themes that already existed. Stage 4 integrated the themes and deliberately searched for material the framework could not accommodate; two areas of misfit were found and are reported (Sections 3.2.2, 3.3.2 and 4.2). |
| 27 | Software | What software was used to manage the data? | Section 2.8. NVivo 12 (QSR International) for code management, memo writing and thematic organisation; substantive analytic work was conducted through manual annotation and team discussion. |
| 28 | Participant checking | Did participants provide feedback on the findings? | Section 2.10. Six purposively selected participants, representing a range of nationalities, genders, tenure and facility types, reviewed an eight-page preliminary thematic summary. All six confirmed the themes were recognisable in their own experience; two suggested refinements to theme wording, which were incorporated. |
|  | ***Reporting*** |  |  |
| 29 | Quotations presented | Are participant quotations presented to illustrate the themes? Is each identified? | Section 3. Illustrative quotations appear throughout and are attributed to de-identified participant codes (P1–P17). To limit deductive disclosure, no quotation is attributed to a combination of participant code, nationality, hospital, specialty and tenure (Section 2.11). |
| 30 | Data and findings consistent | Is there consistency between the data presented and the findings? | Table 2 reports participant counts for all three themes and all eleven subthemes; the same counts appear in the Section 3 headings. A table note explains that theme-level counts denote participants contributing to at least one subtheme, so subtheme counts do not sum to the theme-level count. Site, facility-type and mentorship figures in Table 1 reconcile with each other and with the comparison reported in Section 3.5. |
| 31 | Clarity of major themes | Are major themes clearly presented in the findings? | Sections 3.2–3.4, each with subthemes, participant counts and illustrative quotations, summarised in Table 2. |
| 32 | Clarity of minor themes | Is there a description of diverse cases or discussion of minor themes? | Section 2.10 reports negative case analysis of two participants (P7, P14) who described unexpectedly smooth adaptation, identifying prior international experience and specialist credentials as boundary conditions. Section 3.2.1 reports the ambiguous cases in which hierarchical deference was read as an endorsed norm rather than constrained voice. Sections 3.2.2, 3.3.2 and 4.2 report the two areas where Edmondson’s framework did not accommodate the data. |
